# Development and Validation of an Open Access SNP Array for Nile Tilapia (*Oreochromis niloticus*)

**DOI:** 10.1534/g3.120.401343

**Published:** 2020-06-12

**Authors:** Carolina Peñaloza, Diego Robledo, Agustin Barría, Trọng Quốc Trịnh, Mahirah Mahmuddin, Pamela Wiener, John A. H. Benzie, Ross D. Houston

**Affiliations:** * The Roslin Institute and Royal (Dick) School of Veterinary Studies, University of Edinburgh, Midlothian, EH25 9RG, United Kingdom,; ^†^ WorldFish, Penang, 10670, Malaysia, and; ^‡^ School of Biological, Earth and Environmental Sciences, University College Cork, Cork, T12 YN60, Ireland

**Keywords:** GIFT, Abbassa, aquaculture, Nile tilapia, SNP array

## Abstract

Tilapia are among the most important farmed fish species worldwide, and are fundamental for the food security of many developing countries. Several genetically improved Nile tilapia (*Oreochromis niloticus*) strains exist, such as the iconic Genetically Improved Farmed Tilapia (GIFT), and breeding programs typically follow classical pedigree-based selection. The use of genome-wide single-nucleotide polymorphism (SNP) data can enable an understanding of the genetic architecture of economically important traits and the acceleration of genetic gain via genomic selection. Due to the global importance and diversity of Nile tilapia, an open access SNP array would be beneficial for aquaculture research and production. In the current study, a ∼65K SNP array was designed based on SNPs discovered from whole-genome sequence data from a GIFT breeding nucleus population and the overlap with SNP datasets from wild fish populations and several other farmed Nile tilapia strains. The SNP array was applied to clearly distinguish between different tilapia populations across Asia and Africa, with at least ∼30,000 SNPs segregating in each of the diverse population samples tested. It is anticipated that this SNP array will be an enabling tool for population genetics and tilapia breeding research, facilitating consistency and comparison of results across studies.

Nile tilapia (*Oreochromis niloticus*) is one of the most widely farmed freshwater fish species in the world, with 4.2 million tons being produced in 2016 (FAO 2018). Although this species is native to Africa, Nile tilapia aquaculture has been successfully established in over fifty countries across Asia, Africa, and South America (Eknath and Hulata 2009). The popularity of tilapias stem from their overall ease of culture, which is largely based on their fast growth rate, robustness, relatively short generation interval, and ability to adapt to diverse farming systems and habitats (Ng and Romano 2013; Eknath *et al.* 1998), although see Jansen *et al.* (2019) for discussion of recent disease outbreaks. These attributes make Nile tilapia a suitable species for use in the diverse and often suboptimal farming systems of many low and middle-income countries, where it represents an important source of animal protein and social well-being (Ansah *et al.* 2014).

Several selective breeding programs have been established for Nile tilapia (Neira 2010), among which a major success story is the development of the widely farmed Genetically Improved Farmed Tilapia (GIFT) strain. The GIFT base population was formed in the early 1990s and was composed of eight unrelated strains: four wild populations from Africa (Egypt, Ghana, Kenya and Senegal) and four widely farmed Asian strains (Israel, Singapore, Taiwan and Thailand) (Eknath *et al.* 1993). The main breeding objective of the GIFT program was to improve growth rate, but other relevant traits such as overall survival, resistance to diseases, and maturation rate were also considered (Eknath and Acosta 1998; Trọng *et al.* 2013; Komen and Trọng 2014). Breeding programs have achieved significant genetic gains for growth-related traits in this species. For instance, after five generations of selection the GIFT strain showed a cumulative genetic gain of 67% for body weight at harvest compared to the base population (Bentsen *et al.* 2017). Most of the genetic progress achieved to date for tilapia was obtained through traditional pedigree-based approaches. The use of genome-wide genetic markers to estimate breeding values for selection candidates via genomic selection (Meuwissen *et al.* 2001; Sonesson and Meuwissen 2009) has the potential to increase genetic gain, particularly for traits that are difficult or expensive to measure directly on the candidates. Therefore, the development and application of high density genotyping platforms would be advantageous in expediting genetic improvement in breeding programs for Nile tilapia.

SNP arrays are powerful high-throughput genotyping tools that are increasingly becoming available for aquaculture species including Atlantic salmon (*Salmo salar*) (Houston *et al.* 2014; Yáñez *et al.* 2016), common carp (*Cyprinus carpio*) (Xu *et al.* 2014), rainbow trout (*Oncorhynchus mykiss*) (Palti *et al.* 2015), Pacific (*Crassostrea gigas*) and European (*Ostrea edulis*) oysters (Lapègue *et al.* 2014; Qi *et al.* 2017; Gutierrez *et al.* 2017), catfish (*Ictalurus punctatus* and *Ictalurus furcatus*) (Liu *et al.* 2014; Zeng *et al.* 2017;), Arctic charr (*Salvelinus alpinus*) (Nugent *et al.* 2019), tench (*Tinca tinca*) (Kumar *et al.* 2019), and indeed Nile tilapia (Joshi *et al.* 2018; Yáñez *et al.* 2020). Compared to other high-throughput genotyping methods, such as RAD-Seq (Baird *et al.* 2008), SNP arrays have the advantage of increased genotyping accuracy and SNP stability, as the same markers are interrogated each time (Robledo *et al.* 2018a). These platforms have been used to study the genetic architecture of diverse production traits such as growth (Tsai *et al.* 2015; Gutierrez *et al.* 2018) and disease resistance (Tsai *et al.* 2016; Bangera *et al.* 2017; Robledo *et al.* 2018b), and their utility for genomic prediction in several aquaculture species has been clearly demonstrated (for a review see Zenger *et al.* (2019)).

The two Nile tilapia SNP arrays developed to date are both focused on the broodstock strains of specific commercial breeding programs. One of the platforms was designed based on the analysis of the GenoMar Supreme Tilapia (GST) strain (Joshi *et al.* 2018), whereas the other platform derived from the evaluation of two strains belonging to Aquacorporación Internacional (Costa Rica) and a GIFT population from AquaAmerica (Brazil) (Yáñez *et al.* 2020). These SNP arrays have been shown to be highly effective in the discovery populations, and have been used to generate high-density linkage maps and perform tests of genomic selection (Joshi *et al.* 2020; Yoshida *et al.* 2019a). However, while all of these commercial strains are related to the GIFT strain (which underpins a large proportion of global tilapia aquaculture), their utility and performance in other farmed tilapia strains, especially those inhabiting Asia and Africa, is unknown. To develop platforms that are not exclusively informative in a focal strain, ideally additional SNP panels derived from genetically diverse populations should be evaluated during the SNP selection process (Montanari *et al.* 2019). This strategy would allow to mitigate ascertainment bias, and thus broaden the applicability of a SNP array.

The aim of this study was to develop a publicly available, open access ∼65K SNP array for Nile tilapia based on the widely cultured GIFT strain, but that also contains informative markers in multiple tilapia strains across Asia and Africa. To achieve this, a large SNP database was generated by whole genome Illumina sequencing of pooled genomic DNA from 100 individuals from the WorldFish GIFT breeding nucleus from Malaysia. These newly discovered markers were cross-referenced with SNP panels previously identified in several populations, with the aim of prioritizing markers that are informative across strains. To test the performance of the SNP array, nine Nile tilapia populations of different geographical origins and genetic backgrounds (*i.e.*, GIFT, GIFT-derived and non-GIFT strains / populations) were genotyped. The broad utility and open-access availability of the array is anticipated to benefit both the academic and commercial communities to advance genomic studies in this species and support ongoing and emerging breeding programs.

## Materials And Methods

### Animals, DNA extraction and sequencing

One hundred Nile tilapia broodstock samples from the 15^th^ generation of the core GIFT Nile tilapia-breeding nucleus of WorldFish at the Aquaculture Extension Center in Jitra (Kedah, Malaysia) were used for DNA sequencing for SNP discovery. Caudal fin clips were sampled and preserved in absolute ethanol at -20° until shipment from Malaysia to The Roslin Institute (University of Edinburgh, UK) for DNA extraction, sequencing and genetic analysis.

Genomic DNA was isolated from the tilapia fin clips using a salt-based extraction method (Aljanabi and Martinez 1997). The integrity of the DNA samples was assessed by performing an agarose gel electrophoresis. DNA quality was also evaluated by estimating the 260/280 and 260/230 ratios on a NanoDrop 1000 UV spectrophotometer. The concentration of the DNA extractions was measured with the Qubit dsDNA BR assay kit (Invitrogen, Life technologies). Samples were diluted to 50 ng/ul and then combined in equimolar concentrations to generate two pools of 50 (different) individuals each. Library preparation and sequencing services were provided by Edinburgh Genomics (University of Edinburgh, UK). DNA pools were prepared for sequencing using a TruSeq PCR-free kit (Illumina, San Diego). The two pools were then sequenced at a minimum 90X depth of coverage on an Illumina HiSeq X platform with a 2x150 bp read length.

### SNP discovery in the GIFT strain

The quality of the sequencing output was assessed using FastQC v.0.11.5 (Andrews 2010). Quality filtering and removal of residual adaptor sequences was conducted on read pairs using Trimmomatic v.0.38 (Bolger *et al.* 2014). Specifically, Illumina specific adaptors were trimmed from the reads, leading and trailing bases with a Phred score less than 20 were removed, and reads were trimmed if the average Phred score over four consecutive bases was less than 20. Only read pairs that had a post-filtering-length longer than 36 bp were retained. Cleaned paired-end reads were aligned to the *Oreochromis niloticus* genome assembly published by Conte *et al.* (2017) (Genbank accession GCF_001858045.2) using BWA v0.7.17 (Li and Durbin 2009). To minimize biased estimates of allele frequencies, PCR duplicates were removed from the dataset using SAMtools v1.6 (Li *et al.* 2009). Variants were called from the pools with the software Freebayes v1.0.2 (Garrison and Marth 2012 *preprint*) if (i) at least three reads supported the alternate allele or (ii) the SNP allele frequency in the pool was above 0.02, whichever condition was met first. As a first filtering step, only SNPs that had no interfering variants within less than 40 bp on either side were retained. The resulting vcf file was then filtered to obtain a list of high quality variants with vcffilter v1.0.0 (https://github.com/vcflib/vcflib); bi-allelic SNPs meeting the following criteria were kept for further evaluation: (i) a minimum coverage of 50X and maximum coverage of 150X, (ii) presence of supporting reads on both strands, (iii) at least two reads balanced to each side of the site and (iv) more than 90% of the observed alternate and reference alleles supported by properly paired reads. To enrich the platform for variants located on or nearby genes, polymorphisms were annotated and classified using the software SnpEff v4.3 (Cingolani *et al.* 2012). This list of candidate SNPs were sent as 71-mer nucleotide sequences to ThermoFisher for *in silico* probe scoring.

### Overlap between GIFT SNPs and other datasets

In order to reduce ascertainment bias and increase the utility of the platform across multiple strains, we prioritized markers that also segregated in other strains / populations. The candidate GIFT SNP discovery panel was compared with four other lists of variants. The first panel of variants used for comparison were identified in an inter-generational sample of individuals of the Abbassa strain, a selectively bred Nile tilapia strain from Egypt (Abbassa breeding panel: 6,163 SNPs) (Lind *et al.* 2017). The second SNP panel corresponds to variants discovered in wild fish populations from the region of Abbassa, Egypt (Abbassa wild panel: 6,749 SNPs). The third SNP panel was obtained from a Nile tilapia stock that had been selected for growth for over ten years in Kenya, and that was initially founded by individuals from several populations from East Africa (Kenya breeding panel: 33,085 SNPs). The fourth panel of variants derived from the joint analysis of farmed and wild fish populations from Tanzania (Tanzania panel: 2,182 SNPs). In addition, and as a quality control check, the candidate list of GIFT SNPs was cross-referenced against a panel of markers identified in a sub-sample of the WorldFish GIFT population at Jitra, Malaysia (Wageningen panel: 7,298 SNPs) (Van Bers *et al.* 2012).

### SNP selection

The process of selecting the final panel of SNPs for inclusion on the Applied Biosystems Axiom Tilapia Genotyping Array was as follows. First, SNPs that were previously identified as being associated with phenotypic sex were included (Palaiokostas *et al.* 2013, 2015) (Supplementary Table S1). Second, all SNPs that were shared with at least one other SNP panel – either Abbassa breeding, Abbassa wild, Kenya breeding, Tanzania or Wageningen – were considered as high priority markers and included directly on the array. Thirdly, variants were selected from the collection of high-quality SNPs discovered in the WorldFish GIFT strain. For each SNP that was submitted for evaluation, ThermoFisher assigns a design score (p-convert value) to both 35 bp probes flanking the variant. Probes with a high p-convert value indicate an assay with a higher probability of SNP conversion. Based on their p-convert value, probes can be classified as either ‘recommended’, ‘neutral’, ‘not recommended’ or ‘not possible’. For downstream analysis, SNPs that had at least one probe that was either ‘recommended’ or ‘neutral’ were retained. Next, SNPs were filtered according to their minor allele frequency (MAF) by removing markers with an average MAF (estimated from the two sequenced pools) < 0.05 or > 0.45. The latter MAF threshold was applied to avoid spurious SNPs resulting from sequence differences between paralogues. Additional criteria for SNP selection included filtering out *A/T* and *G/C* variants, as compared to other polymorphisms they require twice as many assays on a ThermoFisher Axiom platform. From the remaining list of high confidence SNPs identified in the discovery population, polymorphisms located in exons were prioritized. To fill the remaining target of ∼65K, SNPs were selected from those located either within a gene or at most at a 1 kb distance. The strategy of enriching for SNPs on genes was followed because they are more likely to alter protein function, and therefore may have a larger effect on phenotypes compared to variants occurring outside genes (Jorgenson & Witte 2006). To obtain a uniform physical distribution across the Nile tilapia genome, all chromosomes and 130 of the longest scaffolds were divided into 10-kb non-overlapping windows, and the SNP with the highest MAF within each interval was selected for inclusion in the platform. Finally, for 1-Mb regions exhibiting the lowest number of markers, the SNP with the highest MAF was included manually.

### SNP array validation

The ThermoFisher Axiom ∼65K Nile tilapia SNP array designed in this study was tested by genotyping nine Nile tilapia populations of different geographical locations and genetic backgrounds (Table 1). The tested fish belonged to one wild population from Egypt (Abbassa wild) and six genetically improved strains. The evaluated strains were the (i) Genetically Improved Farmed Tilapia (GIFT) (Eknath and Acosta 1998; Eknath *et al.* 1993), (ii) Genetically Enhanced Tilapia-Excellent (GET-EXCEL) (Tayamen 2004), (iii) Brackish water Enhanced Saline Tilapia (BEST) (Tayamen *et al.* 2004), (iv) Freshwater Aquaculture Centre (FAC) selected Tilapia (FaST) (Bolivar 1998), and improved strains from (v) Kenya and (vi) Abbassa (Egypt). For each representative strain, a single population was sampled, with the exception of the GIFT strain, for which three populations from different countries were evaluated: Malaysia (discovery population), Bangladesh and Philippines.

**Table 1:** Origin and observed (Ho) and expected (He) heterozygositites for the Nile tilapia populations used for the validation of the SNP array

Population ID	Genetic background	Type	Origin	Number of samples passing QC filters	He	Ho	95% CI (Ho)
GIFT-Ma*^a^*	GIFT	Domesticated	Malaysia	15	0.337	0.350	0.348-0.352
GIFT-Ba	GIFT	Domesticated	Bangladesh	15	0.334	0.347	0.346-0.349
GIFT-Ph	GIFT	Domesticated	Philippines	15	0.322	0.328	0.327-0.330
GET-EXCEL	GIFT-derived	Domesticated	Philippines	15	0.304	0.325	0.323-0.327
BEST	GIFT-derived	Domesticated	Philippines	14	0.294	0.317	0.316-0.320
FaST	Non-GIFT	Domesticated	Philippines	15	0.243	0.252	0.250-0.254
Kenyan	Non-GIFT	Domesticated	Kenya	15	0.236	0.209	0.207-0.211
Abbassa strain	Non-GIFT	Domesticated	Egypt	13	0.229	0.239	0.237-0.241
Abbassa Wild	Non-GIFT	Wild	Egypt	8	0.220	0.258	0.259-0.264

a discovery population.

In total, 135 individuals, comprising 15 fish of balanced sex ratios in each population, were genotyped by IndentiGEN (Ireland) using the Nile tilapia ∼65K SNP array. To perform a principal component analysis (PCA) on the genome-wide SNP data the following SNPs and samples were retained using PLINK v1.9 (Chang *et al.* 2015): (i) SNPs of the Poly High Resolution class (*i.e.*, high quality markers with three well-resolved genotype clusters) (ii) markers with a call rate > 0.95, (iii) individuals with a call rate > 0.90, and (iv) one SNP of a pair showing high linkage disequilibrium (r^2^ > 0.7). In addition, for individuals sharing greater than 80% of alleles identical-by-state (IBS) with another individual, only one was retained for further analysis. The structure of the 135 individuals genotyped with the SNP array was investigated using the R package LEA (Frichot and François 2015), with the significance of the identified components evaluated with Tracy-Widom statistics (Tracy and Widom 1994).

### Summary statistics of SNPs

The levels of observed and expected heterozygosity (Ho, He) for each Nile tilapia strain / population were calculated, and 95% confidence intervals of Ho estimated based on 1,000 bootstrap replicates. To evaluate the informativeness of the SNPs on the array, markers were classified into five different categories depending on their average MAF: Common (MAF > 0.3); Intermediate (0.3 > MAF > 0.1); Low (0.1 > MAF > 0.05); Rare (MAF < 0.05); and Fixed (MAF = 0).

### Linkage disequilibrium magnitude and decay

The estimate of linkage disequilibrium (LD) was based on a version of the SNP dataset in which all individual and SNP QC filters had been applied (see SNP array validation section), except the removal of markers based on LD. As a pairwise measure of LD, r^2^ (Hill and Robertson 1968) was chosen because it is most frequently used in the context of association mapping (Ardlie *et al.* 2002). Moreover, other LD metrics such as D’ are highly affected by sample size (McRae *et al.* 2002) and its use is not recommended when sample sizes are small. LD was estimated separately for each strain / population as the inter-marker Pearson’s squared correlation coefficient r^2^ corrected for relatedness (r^2^vs) using the package LDcorSV v1.3.1 (Mangin *et al.* 2012) in R v 3.5.0 (R Core Team 2014). For comparison, two MAF thresholds were applied to the data before measuring the extent of LD, MAF > 0.05 and MAF > 0.1. The average r^2^ was calculated in 10-kb bins (pairwise distance between SNPs) for each Nile tilapia chromosome. The LD decay was visualized using the R package ggplot2 (Hadley 2009) by plotting the average r^2^ within each bin (across all chromosomes) against inter-marker distances, which extended from ten up to 10,000 kb.

### Ethics statement

Data collection and sampling of the GIFT tilapia populations was performed as part of a non-profit selective breeding program run by WorldFish. The animals from this breeding population are managed in accordance with the Guiding Principles of the Animal Care, Welfare and Ethics Policy of WorldFish. Tissue sampling was carried out in accordance with the norms established by the Reporting *In Vivo* Experiments (ARRIVE) guidelines.

### Data availability

Raw sequence reads from the two pools analyzed for SNP discovery have been deposited in NCBI’s Sequence Read Archive (SRA, https://www.ncbi.nlm.nih.gov/sra) under accession number PRJNA520791. Genome position and probes for all SNPs included in the ∼65K SNP array are given in File S1. The genome positions and allele frequencies of the SNPs included on the array can be found in the European Variation Archive (EVA, https://www.ebi.ac.uk/eva/) under accession number PRJEB38548. The tilapia SNP array is available for commercial purchase from ThermoFisher (array number 551071, E-mail: BioinformaticsServices@thermofisher.com). Supplemental material available at figshare: https://doi.org/10.25387/g3.12472121.

## Results

### SNP selection and array development

The pooled DNA sequencing resulted in 458M and 461M paired-end reads for the two DNA pools. The alignment of the quality control filtered reads against the Nile tilapia reference genome (Genbank accession GCF_001858045.2) led to the discovery of ∼20 million putative polymorphisms. Of the 1,166,652 bi-allelic SNPs that remained after applying post-alignment quality control (QC) filters, 694,348 fell within genes or in the neighboring regions of genes (*i.e.*, within < 1 kb). After additional filtering criteria related to allelic frequency thresholds (removal of SNPs with average MAF < 0.05 or > 0.45) and the type of allele polymorphism (removal of *A/T* and *G/C* variants), 351,188 SNPs were sent as 71-mer nucleotide sequences to ThermoFisher for *in silico* probe scoring. From the list of scored SNP probes provided by ThermoFisher, only those that were categorized as either ‘recommended’ or ‘neutral’ were selected.

The final ∼65K SNP array contained (i) 7 sex determination markers, (ii) 6,883 SNPs discovered in our population that overlap with SNP panels identified in other strains / populations, (iii) 11,328 SNPs located in exons, and (iv) 47,239 SNPs occurring in genes or within < 1 kb of genes. The latter set of SNPs were selected to be evenly spaced according to physical distance along the 22 chromosomes (Supplementary Figure S1) and 130 of the longest unplaced scaffolds of the Nile tilapia genome assembly.

### SNP array validation

After QC of the genotyping data, seven, two and one fish were removed due low call rate from the Abbassa wild, Abbassa strain and BEST population, respectively. Therefore, 125 individual fish from across nine different strains / populations were used to validate the SNP array (Table 1). The obtained raw intensity files were imported to the Axiom Analysis Suite software v2.0.035 for quality control analysis and genotype calling. Genotypes were called following the Best Practices Workflow using the default settings for diploid organisms (Thermo Fisher Scientific Inc 2018). The SNP probe sets were classified into one of the following six category classes based on cluster properties and QC metrics: PolyHighResolution, NoMinorHom, MonoHighRes, Off-Target Variant (OTV), CallRateBelowThreshold, and Other. Of the 65,450 SNPs assayed by the platform, 54,604 SNPs (83.4%) were classified as PolyHighResolution markers, the class with the highest quality probes and presence of both the major and minor homozygous clusters. The number of SNPs that showed a good cluster resolution but no evidence of individuals with minor homozygous genotypes (NoMinorHom) was 2,122 (3.2%). Only 374 SNPs (0.5%) on the array were monomorphic (MonoHighResolution). Among the SNPs that failed to provide reliable genotypes at default settings, 194 SNPs (0.2%) were OTV, 3,026 SNPs (4.6%) had a SNP call rate below the chosen threshold of 0.97 (CallRateBelowThreshold), and 5,130 (7.8%) were not classified into any of the above categories (Other). After applying standard QC filters, 54,310 (MAF > 0.05) and 49,429 (MAF > 0.1) SNPs and 125 individuals were retained for the assessment of LD decay; after the pruning of markers based on LD, 42,460 SNPs remained for the estimation of general population statistics and population structure.

### Minor allele frequency and genetic diversity in Nile tilapia populations / strains

The average observed heterozygosity of the genotyped populations was 0.29, with the GIFT strain from Malaysia (*i.e.*, the primary discovery population) having the highest value (0.35), and the Kenyan population the lowest (0.21) (Table 1). Overall, the observed heterozygosities (Ho) were slightly higher than expected (He), and showed a similar pattern across populations. The only exception was the Kenyan strain, for which the Ho was lower than the He (0.21 *vs.* 0.24).

**Figure 1:**
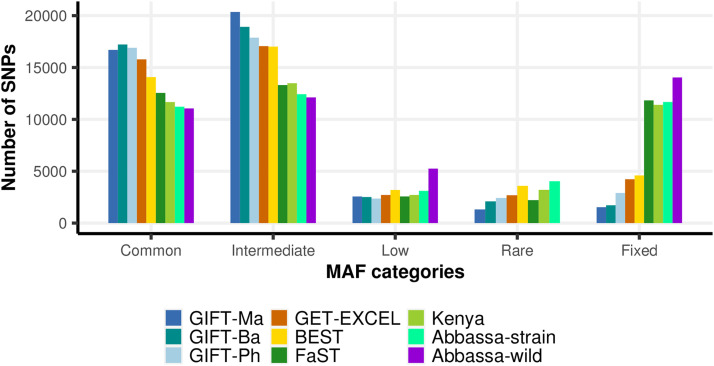
MAF categories of SNPs from the ∼65K SNP-chip across nine different Nile tilapia strains / populations.

**Figure 2:**
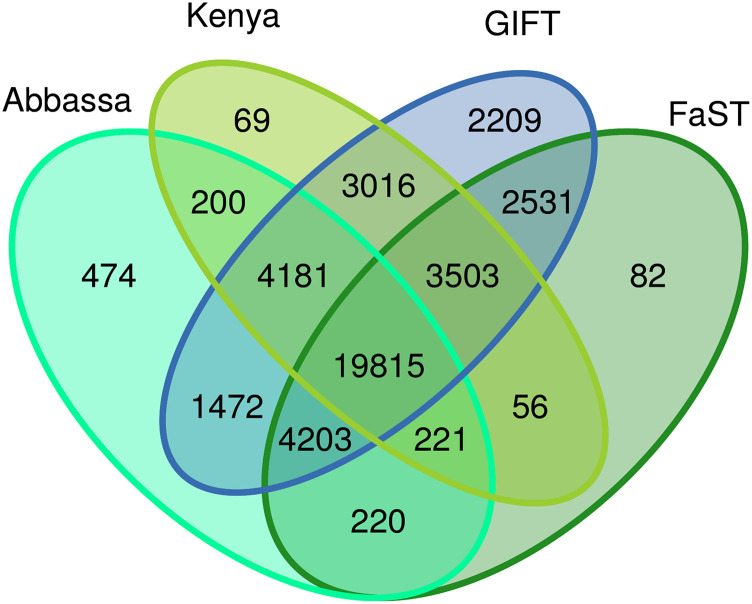
Number of informative SNPs (MAF > 0) shared among the four distinct strains evaluated in this study: Abbassa, Kenya, GIFT and FaST.

The average MAF of all 42,460 successfully genotyped SNPs ranged from 0.23 to 0.26 across the genetically improved strains and the single wild population evaluated. The number of informative markers (MAF > 0) on the array was higher for samples from GIFT and GIFT-derived populations than for populations with non-GIFT genetic backgrounds (Figure 1). The primary discovery population had the greatest number of informative markers, 40,930 SNPs (96%). As expected, the populations genetically closer to the GIFT discovery population from Malaysia had the second and third highest numbers of informative markers – 40,743 (95%) and 39,562 (93%) informative SNPs in the GIFT stocks from Bangladesh and the Philippines, respectively. Likewise, GIFT-derived strains contained a high proportion of informative SNPs, with 38,232 (90%) markers segregating in the GET-EXCEL and 37,867 (89%) in the BEST strain. The number of informative markers for the three non-GIFT strains evaluated in this study were 30,631 (72%) for the FaST strain, 31,061 (73%) for the Kenyan domesticated line and 30,786 (72%) for the Abbassa strain. A large fraction of these informative SNPs co-segregate with the GIFT strain (Figure 2). The average MAF for the markers that are common to all the different representative strains evaluated (total = 19,815 SNPs) was similar and ranged from 0.26 to 0.28. The single wild population analyzed, Abbassa-wild, exhibited the lowest number of informative markers (28,421 SNPs; 66%).

### Population structure

**Figure 3:**
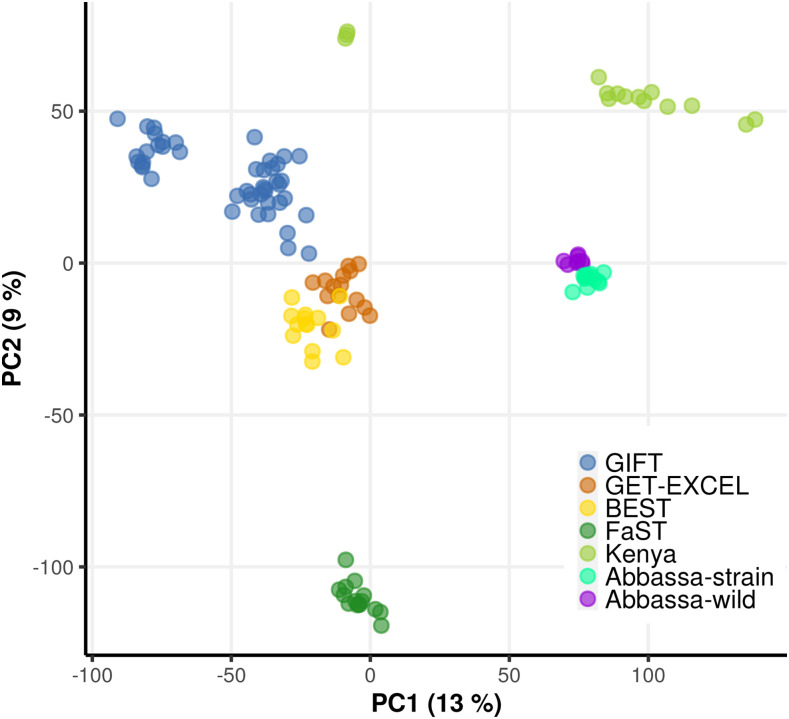
PCA representing the structure of different strains / populations used for the validation of the ∼65k SNP array. The total number of individuals (dots) is 125. Each dot is color coded according to its origin, as shown in the legend at the bottom right corner of the plot.

The population stratification of the nine Nile tilapia strains / populations was visualized using a PCA to reduce the dimensions of the genotype data (Figure 3). The two first eigenvectors accounted for 22% of the total variance. The first dimension, which explains 13% of the variance, mainly separates GIFT and GIFT-derived populations from the Nile tilapia strains / populations of African origin (Abbassa-strain, Abbassa-wild and Kenya). The second principal component explains 9% of the total variance and separates the strains / populations from Africa into two clusters, one comprised of Nile tilapia individuals from Egypt (Abbassa-strain and Abbassa wild) and the other comprising the Kenyan domestic line. Additionally, this dimension also separates Asian GIFT, GIFT-derived and non-GIFT strains into three distinct clusters represented by the (i) FaST strain, (ii) GIFT strains from Malaysia, Philippines and Bangladesh, and (iii) non-GIFT strains, namely GET-EXCEL and BEST. Three individuals of putative Kenyan origin did not group with the Kenyan cluster (those with negative PC1 values in Figure 3).

### Linkage disequilibrium decay

**Figure 4:**
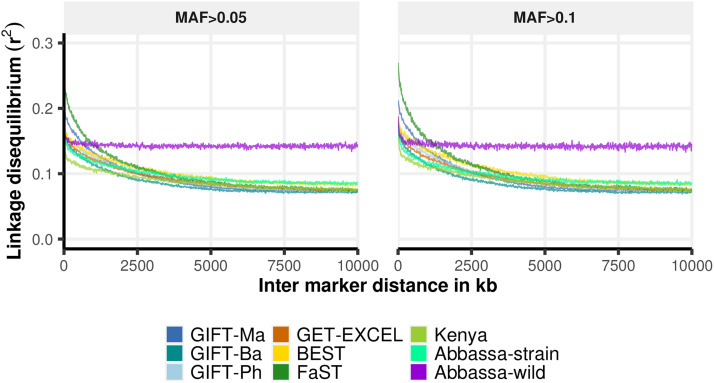
Linkage disequilibrium decay (r^2^) over distance (in kb) among different Nile tilapia strains / populations genotyped with the ∼65K SNP array. LD decay after applying a MAF threshold of 0.05 (left panel) and 0.1 (right panel).

The overall average LD between marker pairs was relatively low and decayed as physical distance increased. Similar patterns of LD decay were observed for the two MAF thresholds, although the MAF filter of 0.1 resulted in higher magnitudes of r^2^ (Figure 4). Two distinct patterns of LD decay were observed across strains / populations. A first group – composed exclusively of domestic lines (GIFT-Ma, GIFT-Ba, GIFT-Ph, GET-EXCEL, BEST, FaST, Kenya and Abbassa-strain) – showed a moderate to low LD decay over both short and long-range distances. The average observed values of r^2^ at the smallest inter-marker distance evaluated (10 kb bin) was ∼0.2 (MAF > 0.1 dataset). Within the short-range distances (< 100 kb), there was a 10–23% decrease in pairwise LD when SNP pairs separated by ∼10 kb were compared to SNP pairs separated by ∼100 kb. Considering long-range distances, the average r^2^ dropped by 65% from that estimated at 10 kb compared to 10,000 kb in GIFT-Ma (0.21 *vs.* 0.08), 60% in GIFT-Ba (0.18 *vs.* 0.07), 61% in GIFT-Ph (0.19 *vs.* 0.07), 59% in GET-EXCEL (0.19 *vs.* 0.08), 54% in the BEST strain (0.19 *vs.* 0.09), 72% in FaST (0.27 *vs.* 0.08), 54% in the Kenyan strain (0.17 *vs.* 0.08) and 52% in the Abbassa-strain (0.18 *vs.* 0.09). In contrast, LD decayed much more slowly in the Abbassa-wild population. The reduction in r^2^ between pairs of SNPs at 10 kb *vs.* 10,000 kb distance apart from each other was only 22% (0.19 *vs.* 0.15) (Figure 4).

## Discussion

The ∼65K SNP array developed in this study is an open-access high-throughput genotyping platform for Nile tilapia. A large majority of the SNPs on the platform were of high quality and polymorphic – 87% of the SNPs fell in either the PolyHighResolution or NoMinorHom categories. This performance value lies in the upper range of current aquaculture SNP arrays (*e.g.*, 89% for rainbow trout (Palti *et al.* 2015) and 77% for the latest catfish array (Zeng *et al.* 2017)), demonstrating the efficacy of our Pool-Seq strategy for robust SNP discovery at a fraction of the sequencing effort of typical SNP chip designs.

Two published SNP arrays have been developed for Nile tilapia, each of ∼58K SNPs (Joshi *et al.* 2018; Yáñez *et al.* 2020). These platforms capture the genetic diversity of specific improved lines, but their efficacy has only been demonstrated in the GST strain (*i.e.*, GIFT line further improved through genomic tools) (Joshi *et al.* 2018) or GIFT and GIFT-related strains from South America (Yáñez *et al.* 2020). In our array, the bulk of SNPs were derived from a SNP discovery process performed on two DNA pools of 100 fish of the core breeding nucleus of the WorldFish GIFT strain, which underpins a large proportion of global tilapia production. However, to mitigate ascertainment bias and widen the applicability of the platform, panels from previous SNP discovery projects were cross-referenced and common SNPs were prioritized. Yet, as expected, the number of informative SNPs decreases with increasing genetic distance from the primary discovery population (*e.g.*, ∼72% across non-GIFT strains; Figure 2). Even though a small number of individuals (∼15 per strain / population) were genotyped with the array, there were at least ∼30,000 SNPs segregating in each of the population samples evaluated, and approximately 20,000 common SNPs segregating in all non-GIFT strains tested, namely Abbassa, Kenya and FaST. Therefore, this SNP array can serve as a common platform for use by the tilapia genetics and breeding community to encourage cross-study comparisons and meta-analyses of genomic datasets.

A principal component analysis demonstrated that our 65K SNP array distinguishes the four major strains evaluated in this study (GIFT, Abbassa, Kenya and FaST), indicating clear independent clusters based on the first two principal components. While the purpose of this analysis was to test the utility of the SNP array to distinguish populations, a few interesting observations were noted. First, individuals from the Abbassa genetically improved strain clustered with wild fish from the same region (*i.e.*, Abbassa, Egypt). This pattern is consistent with a short period of artificial selection that has not yet led to significant shifts in allele frequencies. Additionally, the projection of the Kenyan cluster along a line in the PC plot may indicate the recent admixture of two populations, as suggested for this dispersion pattern by Patterson *et al.* (2006). On the other hand, GIFT and GIFT-derived strains form a loose cluster that separates in dimension 2 of the PC plot but that is not clearly maintained in dimensions 3 to 6 (Supplementary Figures S2-S3). This lack of consistency likely indicates that the population structure of this cluster may not be well represented by the first two PCs. As expected, there is a large degree of overlap among the GIFT strains, most likely due to their common origin. The GIFT-derived strains (GET-EXCEL and BEST) tend to co-cluster in the PC plot; as both strains were developed in the Philippines (Tayamen 2004; Tayamen *et al.* 2004), this concordance could reflect shared breeding goals and similar production systems and breeding practices. Interestingly, although these GIFT-derived strains are the product of selection programs applied to base populations originating from different strains, the PCA suggests they are genetically closer to the GIFT strain. For instance, GET-EXCEL is a synthetic strain developed based on four parental lines: the GIFT strain (8^TH^ generation), the FaST strain (13^TH^ generation), an Egyptian strain (composed by animals sourced from eight locations in Egypt) and a Kenyan strain (coming from stock collected in Lake Turkana) (Tayamen 2004). However, in the PC plot GET-EXCEL individuals group with the BEST strain, closer to the GIFT cluster, and more distant to any of the other strains they supposedly derive from (*i.e.*, Abbassa, Kenya and FaST). This observation may suggest that the GET-EXCEL strain has a limited Abbassa, Kenyan and FaST genetic component, which could be explained by an unequal contribution of parental lines during the establishment of the strain.

Linkage disequilibrium (LD) is the non-random association between the observed frequencies of a particular combination of alleles (Wall and Pritchard 2003). Adequate LD is critical for the implementation of GWAS studies and genomic selection in breeding programs. Both methods exploit the LD that exists between markers and quantitative trait loci (QTL) or causative mutations (Flint-Garcia *et al.* 2003; Goddard and Hayes 2009). Hence, the magnitude and extent of LD decay between genetic markers can be used to predict the marker density required for QTL mapping. For all the evaluated Nile tilapia populations (GIFT, GIFT-derived and non-GIFT), overall relatively low levels of LD (r^2^ ∼0.2) were accompanied by a moderate to slow decay with increasing distance. Despite the small number of animals used to assess LD decay (∼15 individuals per strain / population), a similar pattern was found to that reported by Yoshida *et al.* (2019b) for GIFT and GIFT-derived commercial populations in South America. The weak correlation found between SNPs is consistent with previous findings in GIFT strains (Xia *et al.* 2015; Yoshida *et al.* 2019b) and is comparatively lower than estimates obtained from other farmed fish species such as Atlantic salmon (Barria *et al.* 2018; Kijas *et al.* 2017). Nevertheless, it is worth noting that despite the relatively low levels of LD, the SNP density of the array is in excess of requirements to obtain maximal genomic prediction accuracy in the context of a typical sibling testing breeding program in tilapia (Yoshida *et al.* 2019a), and indeed for the majority of aquaculture species tested to date (Houston *et al.* 2020). Historical factors that affect effective population size (*e.g.*, population bottlenecks, selective breeding) may influence patterns of LD (Gaut and Long 2003). Contrary to the expectation of domesticated lines showing longer LD than wild populations (Gray *et al.* 2009; Whiteley *et al.* 2011), the single wild population examined in this study (*i.e.*, Abbassa wild) showed the slowest rate of decrease and the highest LD at longer distances compared to all Nile tilapia strains evaluated. Because it is possible that this sampled Abbassa population may not be a good representation of wild individuals (*e.g.*, due to interbreeding with escapees) or LD estimates are being biased by population structure (this hypothesis was not tested in this study), additional wild populations should be genotyped and evaluated. The general trend observed across strains of overall low levels of r^2^ suggests that patterns of LD in Nile tilapia are complex and likely associated with particular features of the process of domestication of this species (Xia *et al.* 2015).

At the mean inter-marker spacing on the SNP array (∼16 kb), the average r^2^ across autosomes was 0.2. According to the simulations performed by Hu and Xu (2008), an r^2^ of at least 0.2 is required to achieve a power above 0.8 to detect a QTL for a complex trait of low heritability (h^2^ ∼0.05). Although overall LD levels appear to be low in Nile tilapia, our preliminary results suggest that this array provides sufficient genomic resolution to capture association signals in different strains, and will therefore contribute to expand genetic research in this species and effectively support ongoing and future breeding programs.

## Conclusion

A high quality Nile tilapia SNP array was created and validated in several strains. The SNP array was built by prioritizing markers that are evenly spaced across gene entities and their local neighborhood (within < 1 kb), thereby potentially increasing the chance of detecting variants that alter gene expression and / or protein function. The open-access nature of the SNP array together with demonstration of its utility across multiple strains will facilitate its use in genetic research in this species. This may include studies to assess the origin of farmed populations, to track introgression of farmed genomes into the wild, and to understand the genetic architecture of traits of interest. Further, this SNP array will contribute to the management of farmed tilapia populations, and enable accelerated genetic gain and better control inbreeding in breeding programs via genomic selection.
